# Emerging point‐of‐care autologous cellular therapy using adipose‐derived stromal vascular fraction for neurodegenerative diseases

**DOI:** 10.1002/ctm2.1093

**Published:** 2022-12-10

**Authors:** Rawan Al‐kharboosh, Jonathan Jude Perera, Alexandra Bechtle, Guojun Bu, Alfredo Quinones‐Hinojosa

**Affiliations:** ^1^ Department of Neuroscience Mayo Clinic Jacksonville Florida; ^2^ Department of Regenerative Sciences Mayo Clinic Graduate School Rochester Minnesota; ^3^ Department of Neurosurgery Mayo Clinic Jacksonville Florida; ^4^ Department of Biomedical Engineering Duke University Durham North Carolina

**Keywords:** adipose tissue, neurodegeneration, stem cells, stromal vascular fraction, SVF, therapy MSC

## Abstract

Neurodegenerative disorders are characterized by the gradual decline and irreversible loss of cognitive functions and CNS structures. As therapeutic recourse stagnates, neurodegenerative diseases will cost over a trillion dollars by 2050. A dearth of preventive and regenerative measures to hinder regression and enhance recovery has forced patients to settle for traditional therapeutics designed to manage symptoms, leaving little hope for a cure. In the last decade, pre‐clinical animal models and clinical investigations in humans have demonstrated the safety and promise of an emerging cellular product from subcutaneous fat. The adipose‐derived stromal vascular fraction (SVF) is an early intervention and late‐stage novel ‘at point’ of care cellular treatment, demonstrating improvements in clinical applications for Multiple Sclerosis, Alzheimer's disease, and Parkinson's disease. SVF is a heterogeneous fraction of cells forming a robust cellular ecosystem and serving as a novel and valuable source of point‐of‐care autologous cell therapy, providing an easy‐to‐access population that we hypothesize can mediate repair through ‘bi‐directional’ communication in response to pathological cues. We provide the first comprehensive review of all pre‐clinical and clinical findings available to date and highlight major challenges and future directions. There is a greater medical and economic urgency to innovate and develop novel cellular therapy solutions that enable the repair and regeneration of neuronal tissue that has undergone irreversible and permanent damage.

## Graphical abstract

- Adipose‐derived stromal vascular fraction (SVF) is an emerging autologous cellular therapeutic which mediates repair through ‘bi‐directional’ communication in response to pathological cues.
- SVF maintains a high safety index in pre‐clinical and clinical investigations of multiple sclerosis, Alzheimer's disease and Parkinson's disease. Pre‐clinical evidence suggests that SVF modulates the inflammatory response via macrophage and T‐cell polarization locally and systemically.
- SVF‐treated groups result in significantly better outcomes compared toits purified and cultured mesenchymal stromal cell/adipose stem cell counterpart, improving behavior and disease severity at early intervention time‐points.
- Outcomes of SVF cellular therapy is dependent on SVF composition, which widely differs from patient to patient.

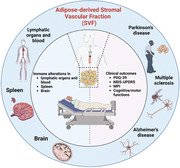

## 1 BACKGROUND

A dearth of measures tha hinder progressive neurodegeneration and enhance neural recovery force patients to settle for managing gradually worsening acute symptoms, leaving little hope of a return to normalcy. Neurodegenerative diseases contribute to one of the most significant health issues among the aging population. In the United States, 5 million people are suffering from Alzheimer's disease (AD); 1 million from Parkinson's disease (PD); 400 000 from multiple sclerosis (MS); and 30 000 from Amyotrophic lateral sclerosis (ALS) and Huntington's disease (HD).^1^, ^2^ Parkinson's alone substantially hampered the US economy, with an estimated $51.9 billion price tag in 2017, while Alzheimer's cost an exorbitant $305 billion in 2020 and is estimated to rise to an alarming $1 trillion by 2050.^3^ Globally, the number of people over 60 is expected to double from 962 million (2017) to 2.1 billion by 2050.^4^ With an aging population, neurodegenerative diseases are projected to rapidly increase in the upcoming years, leaving a large segment at greater risk and with minimal treatment options.^3^

The primary mechanism underlying neurodegeneration is the progressive decline in function, structure, and role of neuronal and glial activity, resulting in permanent loss or eventual cell death, partly ascribed to a hyperactive inflammatory milieu.^5^ Not surprisingly, aging alone exhibits similar immunological properties as neurodegenerative pathologies defined by enhanced mechanisms to clear accumulated protein aggregates, misfolded proteins, debris, or other processes that go amiss throughout one's lifetime. However, hyperinflammation and chronic production of cytokines leads to detrimental alterations of central nervous system (CNS) tissue.^6^ Therefore, strategies aimed at controlling the inflammatory response or repairing cells that have undergone damage represents a viable option to replenish, repair, reduce, or delay degeneration. Strategies using cell therapies largely focus on the replenishment of dying or damaged neuronal populations, immuno‐modulation and re‐wiring of resident neural stem cells.^7^

## 2 CELLULAR THERAPY FOR NEURODEGENERATIVE DISEASES

The field of cellular therapy has utilized the applications of neural stem cells (NSCs),^8^, ^9^, ^10^, ^11^, ^12^, ^13^ induced pluripotent stem cells (iPSCs)^14^, ^15^, ^16^ and mesenchymal stromal cell (MSCs)^17^, ^18^, ^19^, ^20^ as candidates to slow the progression of neurodegenerative diseases. Such applications mediate repair through anti‐apoptotic, anti‐fibrotic, angiogenic and immune‐modulatory processes while accelerating endogenous repair with minimal to negligible adverse events.^21^

Advances in stem cell applications offer hope for patients when available treatments (surgical and pharmacological) fail to arrest the progressive and irreversible consequences of degeneration.^22^, ^23^, ^24^ The therapeutic benefits associated with cell therapy are ‘neuroprotection’ through paracrine signaling and the secretion of growth factors to ‘jump‐start’ the niche for renewal.^25^ However, major challenges with regenerative applications using MSCs, NSCs or iPSCs are the generation of sufficient volumes of viable cells, enhanced metabolic activity, sustained cellular communication, and clinically relevant cell systems that mimic the tissue stroma at site of implantation.^23^, ^26^ Though there are advantages to allogenic cell products such as reduced harvesting variability and donor incompatibilities,^27^ disadvantages include practical complexities related to donor health, immuno‐rejection, cost, time and identity (Table 1).^28^, ^29^, ^30^, ^31^

**TABLE 1:** Comparing cellular applications currently used for neurodegenerative disease. Advantages and disadvantages of the stromal vascular fraction (SVF) against mesenchymal stromal cells (MSCs); neural stem cells (NSCs) and induced pluripotent stem cells (iPSCs)

	Advantages	Disadvantages
**SVF**	Same‐day acquisition and administration No histocompatibility barriers (alw autologous) Heterogeneous Physiologically relevant Abundant (adipose tissue) High safety index Minimal toxicity Multiple cellular subsets	Field and application in its infancy Short duration/retention Variable efficacy across patients Requires multiple injections Extent of prolonged efficacy unknown Little known about impact of age, sex, co‐morbidities on product potency
**MSC**	Standardization (need for surrogate markers identifying stromal and/or stem subpopulations) Allogenic and/or autologous Culturally expandable Hypoimmunogenic Highly characterized and studied Good safety index Tropic to areas of injury in CNS Capacity to differentiate in situ	Short duration (∼2‐4 weeks) Variable efficacy across studies Low retention Stem cell/self‐renewal capacity variable Requires multiple injections Manufacturing time Moderately costly
**NSC**	Notable outcomes for CNS disorders across literature Efficient CNS integration/differentiation Replenish stem reservoir Physiologically relevant Well characterized and studied	Fetal source Potential immunogenicity Ethical concerns Non‐abundant Source prohibitive Very costly Requires viral transformation Long manufacturing time
**iPSCs**	Patient as the optimal source can be extremely informing Expandable/replenishable Reprogrammable cells/pluripotent Alternative source to embryonic stem cells	Ethical concerns Concerns of oncogenic transformation due to ectopic oncogene transcription Transduction efficiency low Integration of viral DNA into host genome (anon‐integrative methods are being tested) Extreme regulatory hurdles due to high safety concerns Extremecostly Exhaustive manufacturing pipeline Potential immunogenicity

MSCs are widely acknowledged as hypoimmunogenic with a high safety index due to their limited MHC I and co‐stimulatory molecules, making them amenable to allogenic transplantation. MSCs slow neuronal cell death with demonstrated alleviation of clinical manifestations of pathology related to motor symptoms,^32^ though studies have demonstrated rejection, suggesting that MSCs may not be fully immuno‐privileged.^33^ Despite their demonstrated safety, their short lifespan in vivo makes their therapeutic impact transient.^34^ NSCs require fetal cells or viral transformation from sources like iPSCs or embryonic stem cells to generate enough cells for application, though their neurotrophic production^12^ and neuroplasticity make them ideal candidates for CNS transplantation in situ.^13^ iPSCs are ideal candidates for cell specificity, having demonstrated full tissue integration and improvements of functional outcomes,^16^ yet requires genetic alterations to expres Yamanaka factors (OCT4, SOX2, KLF3 and C‐MYC) for transformation. Such reprogramming poses risks for oncogenic activation and genetic instability.^35^ It is reasonable to explore alternative applications that do not require genetic transformation, skilled‐personnel or long‐term culturing. Therefore, we propose adipose‐derived stromal vascular fraction (SVF), as an alternative source of cell‐based therapy.

## 3 AUTOLOGOUS POINT‐OF‐CARE SVF CELLULAR APPLICATION

The issues of cellular therapy are being addressed by recent applications of SVF for neurodegenerative diseases.^26^ SVF, defined as adipose derived cells from the stroma of white adipose tissue of subcutaneous fat, is obtained immediately after fat harvesting. Unlike MSCs, iPSCs, or NSCs, SVF does not require culture expansion and can be prepared for administration within hours of lipoaspiration. Ease of patient harvest, abundance of tissue, and relevant immune constituents in SVF make it an attractive alternative therapy for diseases requiring dynamic and rapid response to reduce the inflammatory cascade while mitigating tissue repair.

**FIGURE 1:**
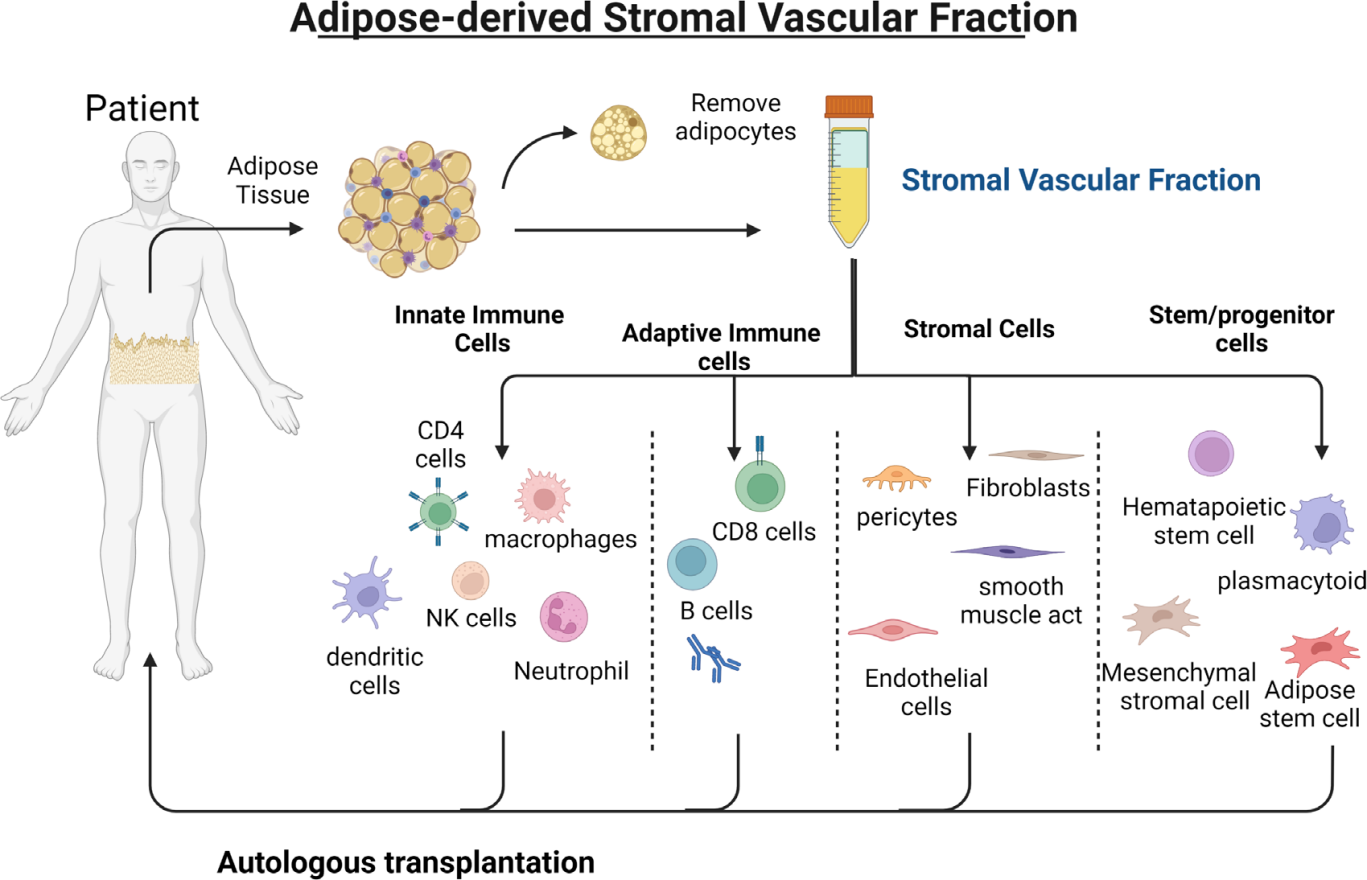
Proposed cellular constituents comprising adipose‐derived stromal vascular fraction (SVF). A breakdown of the SVF into four primary cellular components: *Innate immune* fraction comprising antigen presenting cells; *adaptive immune cells* capable of cytolytic activity and immunological memory; *stromal cells* that provide structural repair to maintain tissue integrity; and *stem/progenitor cells* for regeneration, homeostasis, and cellular reconstitution

SVF is a heterogeneous cellular fraction forming a robust ecosystem comprised of haematopoietic, fibroblast, endothelial, pericytes, adipose‐derived progenitor (ADSCs) and MSCs,^26^, ^36^, ^37^ serving as a valuable source of autologous therapy for diseases where effector cells must bypass major histocompatibility barriers^36^ (Figure 1). Adipose tissue, composed of mature and premature progenitor cells, give rise to several cell types and have widely been used for regenerative applications,^38^ tissue repair, angiogenesis^39^ and immune‐modulation.^40^, ^41^ Studies demonstrate SVF's utility for multiple conditions across several connective and supportive tissue types .^42^ Early investigations have demonstrated its capacity to alleviate disease progression by modulating immune activity for neuroprotection. While manufactured and expanded stem cell applications have shown promise, treatments are primarily confined to improving symptoms rather than slowing down, repairing, or preventing further damage.^43^, ^44^ It is vital to develop therapeutic strategies for neurodegenerative diseases that can *simultaneously* target multiple purported degenerative mechanisms by controlling chronic immune processes,^45^ production of autoreactive antibodies, and stabilizing lymphocyte activity to promote immune tolerance. These goals require innovative strategies beyond the conventional approaches. Such requirements point to the SVF as an alternative cellular therapeutic.

Immune and stem cells are cells of ‘repair’; their activity is strongly pathology‐dependent and highly receptive to minimal changes to the tissue environments in which they are implanted. Therefore, we hypothesize that the added advantage of stem cells and immune cells found in one fraction, as in the SVF, offers a physiologically relevant ‘cell‐communication’ paradigm acting in synchrony to modulate tissue stroma according to specific tissue requirements.

To date, SVF applications lack robust evaluation of the bioactive components mediating therapeutic benefits, yet its clinical potential is currently being explored. The utility of SVF therapy is derived from the multitude of cell types capable of behaving in a coordinated manner, ultimately adapting to the host environment to accelerate repair. Physiologically, tissue regeneration and repair are permitted by a heterogeneous cellular milieu, such as that of the SVF, to synchronize in order to repair pathological aberrations that are partly exacerbated by a hyperactive immune response during aging and neurodegeneration.

## 4 INFLAMMATION IN NEURODEGENERATION AND AGING

Age‐related inflammation is implicated in the progression of neuronal degeneration. Effects of dysregulated immune surveillance contribute to age‐related pathologies widely presumed to be due to a low, yet chronic, activation of innate immunity – a phenomenon also known as ‘inflammaging’.^46^ Overactive immune mechanisms result in the hyperproduction of pro‐inflammatory cytokines leading to detrimental age‐related pathologies accompanied by progressive loss in mental, cognitive, and motor abilities.

Moreover, neurodegenerative diseases share a hallmark event marked by the aggregation of misfolded proteins. In ALS, ubiquitinated inclusions of mutant TDP‐43 are cytoplasmically redistributed,^47^ while MS upregulates components of the endoplasmic reticulum response due to stresses from misfolded protein accumulation.^48^ In Parkinson's, protein filaments of α‐synuclein fibrils accumulate into Lewy bodies and neurites causing cell death of dopaminergic neurons.^49^ Similarly, abnormal aggregation of hyperphosphorylated tau (neurofibrillary tangles) or the breakdown of amyloid precursors (β‐amyloid) form neurotoxic plaques in AD and PD.^50^, ^51^ Consequently, resistance of aggregates to protease degradation give rise to chronic inflammation to clear misfolded proteins and reduce damage to neurons and surrounding glia.^5^

The accumulation of misfolded proteins lead to acquired and progressive chronic activation of immune cells resulting in elevated levels of inflammatory molecules in the local niche.^52^ To clear misfolded proteins, persistent inflammatory signals eventually result in local cell and tissue degradation. Aging‐associated neurodegenerative diseases such as MS, ALS, PD and AD would benefit from therapeutic approaches that ameliorate, neutralize or suppress the heightened inflammatory response. The SVF represents a robust cellular composition of dynamic processes shown to influence local and peripheral inflammatorymilieu,^19^ and may be the key to unlocking new treatment options that slow or reverse neurodegenerative damage.

### 4.1 Multiple sclerosis

Multiple sclerosis is an autoimmune neurodegenerative disorder, partly characterized by chronic inflammation leading to the consequential demyelination and axonal damage.^53^ This progressive damage results in plaques and lesions and the ultimate t destruction of neuronal tissue.^54^ In parallel, a breakdown of the blood‐brain barrier (BBB) ensu to allow the infiltration of immune cells from the periphery.^55^, ^56^ Consequently, patients experience a progressive decline in physical and cognitive ability. Current therapies (steroids and immune modulators) are largely ineffective and are more focused on alleviating symptoms responsible for long‐term side effects.^57^

### 4.2 Amyotrophic lateral sclerosis

Amyotrophic lateral sclerosis affects the motor functions of the spinal cord and brain, causing progressive muscle weakening, atrophy, and eventual paralysis of voluntary movement.^24^, ^58^ Due to the rapid nature of its progression, median survival ranges from 24 to 48 months.^24^ There have been over 60 molecules tested as potential treatment options for ALS, yet none have demonstrated efficacy in clinical trials.^59^, ^60^ It is demonstrated that systemic and local alterations of T cells exacerbate ALS through the release of TNF‐α, IL‐1β and IFN‐γ, supporting the role of these mediators in disease progression.^61^ In response, resident microglia and infiltrating macrophages become over‐activated, leading to neuroinflammation, oxidative stress and degeneration.^62^

### 4.3 Alzheimer's disease

Alzheimer's disease is the most common cause of dementia, progressively eroding and worsening cognitive abilities, memory, personality, and independent thought which ultimately affects the capacity to lead a normal life. Three core pathologies denote the degenerative mechanism of AD: β‐amyloid plaque deposition, neurofibrillary tangles of hyperphosphorylated tau, and sustained activation of immune response^52^; To clear plaques, tangles, and debris, microglia ramp up surveillance and phagocytic mechanisms, but often such activation also leads to the release of pro‐inflammatory cytokines such as IL‐1,^63^ IL‐6,^64^ MCP‐1 and TNF.^65^ In parallel, NF‐κB transcription factor is activated in response to the pro‐inflammatory cytokine milieu resulting in reactive gliosis and damage to the CNS.^66^, ^67^ Mechanisms that dampen the immune response is expected to slow down disease progression by reducing the core pathologies associated with the loss of neuronal integrity.^68^

### 4.4 Parkinson's disease

Parkinson's disease affects dopaminergic neurons in the substantia nigra and other brain regions.^69^, ^70^ Though its deteriorative effects on motor function have been long observed (‘shaking palsy’), symptoms range from minor mental decline to memory loss to dementia; dementia develops in 25%–75% of PD patients.^70^, ^71^ Treatments include dopamine agonists, deep brain stimulation and physiotherapy.^72^ The consequences of an imbalanced inflammatory response favor the progression of Parkinson's through activated microglia with enhanced MHC‐II, ICAM‐1, LFA‐1 production and MHC‐II overexpression.^73^ Similarly, peripheral macrophages release IL‐12, TNF‐α, INF‐γ to stimulate neurotoxicity,^5^, ^63^ while dendritic cells secrete IL‐1β, IL‐12^73^ at the lesion, triggering neuronal death and reactive gliosis. Cell therapeutics that reduce inflammation and re‐establish homeostasis at the local niche to induce CD4 T‐cell into T regs or Th2 represent a practical approach to reduce disease severity.

**FIGURE 2:**
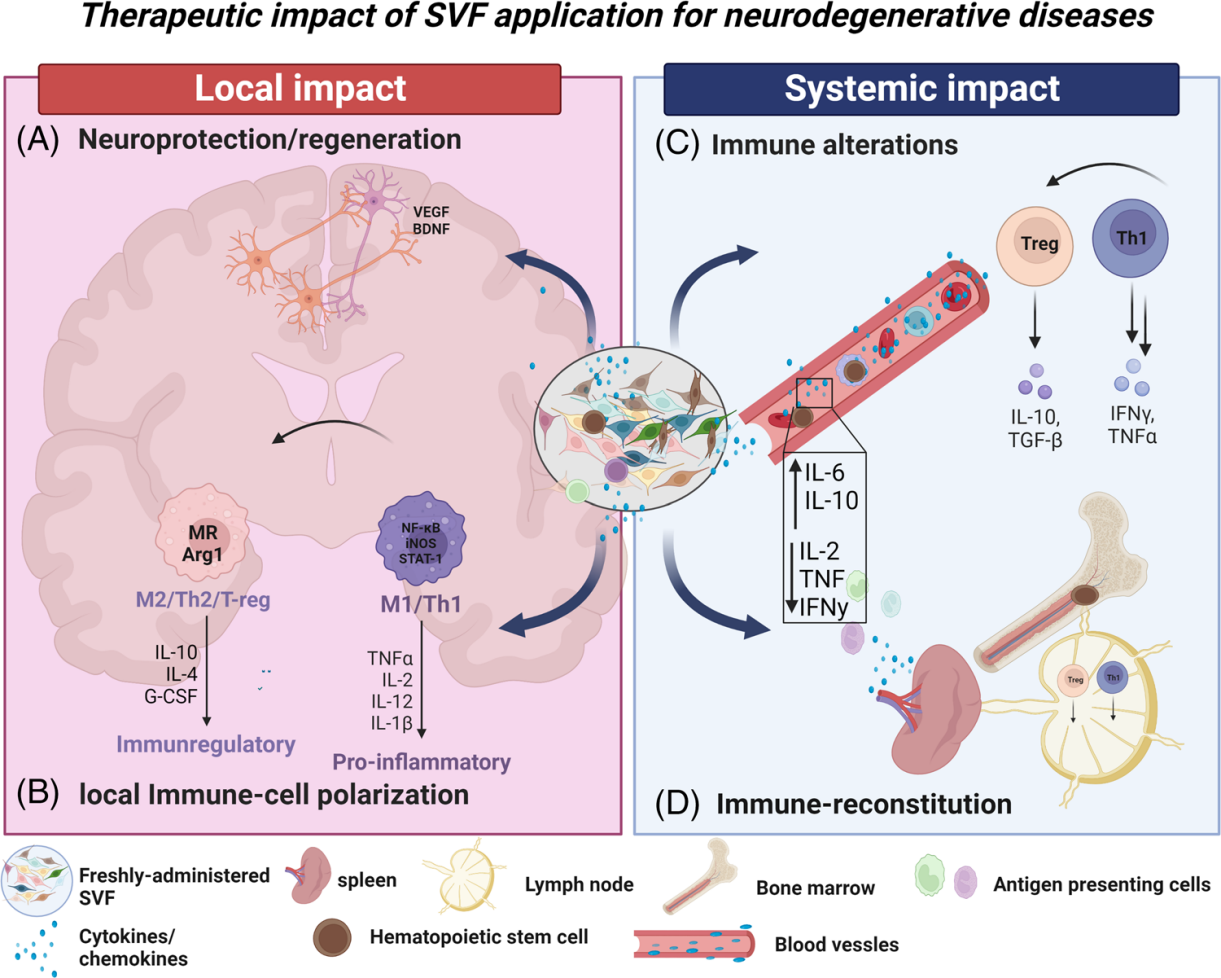
Therapeutic impact of SVF cellular application for neurodegenerative diseases. The SVF imparts its therapeutic impact in the local and distal environments. Local: (A) neuroprotection and regeneration via SVF release of growth and soluble factors. (B) SVF induces polarization of macrophages and T cells to immunoregulatory cells. Systemic: (C) SVF releases anti‐inflammatory factors in the sera which travels systemically and (D) into the spleen and lymph to mediate immune‐regulation and reconstitution. SVF: stromal vascular fraction; VEGF: vascular endothelial growth factors; BDNF: brain‐derived neurotrophic factor; T‐reg: T‐regulatory cells; Th1: T‐helper 1; M1/M2: macrophage‐1/2; G‐CSF: granulocyte colony stimulating factor; TNF: tumour‐necrosis factor; TGF: transforming growth factor; IFN: interferon.

Therefore, there is increased attention in pioneering innovative and alternative therapy routes able to integrate regenerative mechanisms with immunomodulation. Previous studies demonstrate the efficacy of isolated adipose‐derived SVF constituents, such as the MSC or ADSCs, expanded ex vivo to counteract inflammation in autoimmune diseases and enhance neurogenesis.^74^, ^75^ Only recently have studies examined the effects of the crude, freshly extracted, non‐laboratory processed SVF and its ability to attenuate disease severity in the neurological and neurodegenerative space. The proposed processes in pre‐clinical studies suggest several mechanisms by which the SVF acts locally and systemically to mitigate neurodegenerative diseases (Figure 2).

## 5 IMPACT OF SVF TREATMENT ON DISEASE SEVERITY

### 5.1 EAE and MS

SVF has shown promise in murine models of chronic myelin oligodendrocyte (glycoprotein 35–55‐induced) experimental autoimmune encephalomyelitis (EAE),^76^ an established animal model for MS. The study by Semon et al. was preventive in that treatments were administered simultaneously with EAE induction. Murine C57BL/6 received an intraperitoneal injection (IP) of freshly isolated mouse derived SVF compared to its culturally expanded adipose stem cell (ASC) counterpart. SVF and ASC treatment significantly delayed onset and reduced severity scores over the course of 30 days. A significant reduction in the cumulative EAE disease score was observed in the SVF‐treated groups compared to controls (SVF: 9.4; ASC: 13.0; control: 30.4). The SVF‐treated group displayed potent neuroprotective effects; only 25% of SVF‐treated animals demonstrated clinical signs of disease versus 58% from ASC‐treated, against 100% of controls. Similarly, treatment demonstrated a significant reduction in clinical signs of disease onset and severity, resulting in a 75% decrease in SVF incidence compared to controls. Histological analysis revealed both treatment groups reduced demyelination quantified by areas of intact myelin. SVF treatment significantly reduced clinical signs of disease onset and progression compared to expanded ASC. While the mechanism and therapeutic benefits imparted by the SVF are unknown, prevention of chronic EAE by SVF was demonstrated by a strong reduction in axonal damage, demyelination, and spinal cord inflammation. Study authors repeated the same work on human derived SVF, with similar outcomes.^77^

Another study by Bowles et al. evaluated the impact of SVF treatment for early‐stage intervention.^78^ SVF and ASCs were administered 8 days post‐EAE induction (DPI) during pathogenic progression to coincide with early onset of symptoms, motor impairment and early inflammation. Though both SVF and ASC treatments enhanced disease severity by day 12, only the SVF‐treated group significantly reduced severity score with continued progressive improvements 1 week after (15 DPI). Though the SVF‐treated group exhibited the greatest reduction in lesion frequency, it is noteworthy to mention that SVF treatment was also accompanied by a higher lesion surface area. Markedly, SVF‐treated animals only exhibited loss of tail tone, mildly abnormal gate, and lower average disease score, while ASC and control groups displayed severe hindlimb weakness and cognitive impairments. However, from 20–30 DPI, all EAE mice had comparable numbers of rears, implying a wearing down of the initial effects of the SVF dosing.^78^ Overall, the SVF‐treated groups demonstrated superior outcomes than their purified and cultured ASC counterpart, improving behavioral outcomes and disease severity at the early intervention state.

In addition to preventative and early use of SVF in EAE, recent work has demonstrated the therapeutic efficacy of SVF in late‐stage EAE.^79^ Murine SVF administered 20 days post‐disease induction (to mimic late‐stage/severe EAE) were evaluated at 30 days. SVF decreased disease severity and improved behavior and motor function, ultimately resulting in a significant drop in mean clinical score. While all groups were physically impaired by day 20, at day 30, ASC‐ and SVF‐treated mice displayed markedly higher total movement durations and average velocity, suggesting a recovery of motor skills and hindleg strength. Both treatment groups showed histological improvements in levels of myelin in the spinal cord. SVF reduced the size of lesions by > 30% and frequency of lesions by 10%, compared to controls. While the mechanism and therapeutic benefits imparted by the SVF are unknown, prevention, early intervention, and late‐stage disease of chronic EAE were markedly ameliorated. Outcomes were demonstrated by a substantial reduction in axonal damage, demyelination, and spinal cord inflammation.^79^ Elucidating the key molecular players by which SVF imparts its therapeutic benefits is necessary to unravel which component of the SVF fraction is responsible for disease suppression at the later stage.

## 6 SVF TREATMENT MITIGATES THE INFLAMMATORY MILIEU

### 6.1 EAE and MS

In models of EAE and MS, peripheral immune activation is mediated by cytotoxic T cells and macrophage infiltration in the CNS. In the preventive model of EAE,^76^ where disease induction and cellular treatment were administered concurrently, SVF treatment resulted in a marked reduction of inflammatory infiltrates (SVF: 8.5%; ASCs: 7.9%; controls: 14.6%). The reduction of immune cells post‐SVF treatment is postulated to result from diminished IL‐12 and IFN‐γ in sera, indicative of a pathology‐attenuated response and reduced Th‐1.^78^ Reduction of immune cells in the lesions post‐SVF treatment was associated with a drastic delay in disease onset compared to ASCs and untreated controls.

Analysis of CNS tissue post‐SVF treatment at earlier time‐points (6 days post‐treatment) in the EAE model resulted in a two‐fold increase of infiltrating myeloid population (CD11b) and enrichment of anti‐inflammatory macrophages.^78^ Enhancement of M2 immune‐suppressive macrophages (CD11b/CD206) in the CNS suggests that SVF is associated with local and peripheral immune alterations as the first recourse. In a similar study, SVF and ASC treatment increased alternative macrophage frequency around the perivascular areas adjacent to the lesion at day 5 and day 10; only ASC treatment saw a concomitant upregulation of classical (M1) macrophage accumulation (CD11b/CD86).^78^ Another study observed an upregulation of Arginase‐1 (Arg‐1) and nitric oxide synthase (iNOS) gene expression levels in macrophages compared to untreated controls.^80^ Ultimately, SVF induces physiological cues directing macrophage activation and differentiation in the CNS in response to progressive inflammatory aggression. The observed infiltration of alternative macrophage in CNS tissue post‐SVF treatment suggests that SVF readily recruits and polarizes myeloid cells at the lesion to mitigate the inflammatory landscape in EAE. Outcomes were supported by reduction in astrogliosis, suggesting an attenuation of inflammatory mechanisms.^80^

Bowels et al. evaluated the frequencies of infiltrating lymphohaematopoietic cells to elucidate the impact of immune alterations in the CNS post‐SVF treatment. At earlier time‐points, levels of infiltrating T cells, measured by pan CD3, were comparable among all groups. However, a modest increase in mature CD4 cells and CD8 T cells following the SVF treatment group compared to ASC and untreated controls were observed thereafter. Study authors did not clarify the activation state of CD4, therefore, hypothesizing that the impact of such accumulation is limited.

Findings from gene expression analysis revealed that SVF treatment produced the greatest change in cytokine production. IL‐10 and IL‐6 were markedly upregulated in both the brain and spinal cord of SVF mice 10 days after treatment, while INF‐ γ and TNF‐ α were upregulated in the spinal cord only.^79^ Pro‐ and anti‐inflammatory cytokine production induced by SVF may point to biological attempts to restore and maintain balance in the niche. Induction of inflammatory arbitrators, such as IL‐10 and IL‐6, is congruent with clinical studies discussed later in this review where SVF is suggested to promote immune tolerance to manage the body's autoimmune and inflammatory reactions. Consequently, findings demonstrate that SVF polarizes infiltrating macrophage through the induction of IL‐10 and IL‐6.

## 7 SVF‐MEDIATED ALTERATIONS IN LYMPHATIC ORGANS AND BLOOD

In a late‐stage EAE model, it was found that SVF treatment imparted the greatest increase in macrophage, helper T‐cell, and B‐cell populations in the spleen, lymph nodes, and blood, 10 days post‐treatment compared to ASC and vehicle‐treated controls.^79^ Increase in lymphoid cell frequency was associated with enlarged lymph nodes in the SVF‐treated group; no remarkable change was observed between ASC and untreated controls. It is known that cytolytic T cells and reduced T‐reg populations exacerbate EAE pathology. The examined lymph nodes from SVF‐treated groups contained the greatest T‐cell frequency and the lowest cytolytic T‐cell abundance. This suggested an immunological switch supported by an observed reversal of T‐cell abundance upon SVF treatment, which saw increased T‐reg frequency in the lymph nodes 5 days after administration. Similarly, intrasplenic presence of SVF was associated with increased splenic T regs.^80^ Outcomes demonstrate that the SVF reduces disease severity by distally modulating immune subsets under inflammatory conditions. The study further found that the rapid increase of splenic T regs was associated with an induction of alternative splenic macrophages 5 days after treatment.

The proposed mechanism resulting in diminished Th1 frequency enhanced alternative macrophages and T regs accumulation is mediated by SVF production of IL‐10 and TGF‐β. Such cytokines are suggested to promote regulatory T cells and macrophage differentiation in situ.^78^ Similarly, IL‐4 levels were markedly higher in the spleen than PBMCs showing that the SVF mediates immunomodulation at distant organs resulting in better outcomes compared to purified ASCs. In the early interventional study of EAE, spleens from SVF‐treated groups exhibited diminished Th1 and Th17 T cells, with a concomitant increase in anti‐inflammatory cytokines.^78^ Furthermore, IL‐10 was elevated in the spleen and serum of SVF treatment groups. SVF treatment mediated IL‐10 and TGF‐β levels, potentially owing to the enhanced myelin and reduced inflammatory cellular infiltrates compared to ASC treatment and controls.

To evaluate the direct impact of SVF in the spleens, study authors co‐cultured SVF or ASCs and splenocytes (1:1 dose) and measured the level of cytokines. Both SVF and ASCs reduced IL‐2R and incrementally decreased IL‐12, while IL‐23 and IL‐6 were comparable to vehicle‐treated groups.^78^ Additionally, both SVF and ASC significantly reduced IL‐4 and enhanced TGFβ, IL‐10 and Foxp3 in the spleen.^78^ T‐cell‐associated cytokine levels were significantly higher after SVF treatment, demonstrating the superior outcomes induced by the crude fraction compared to purified ASCs. It is unknown whether the observed cytokine levels from co‐cultures are mediated or derived from the SVF itself, as co‐culture assays do not discern the source. Overall, the study demonstrated an SVF‐mediated alteration of T‐cell function and macrophage differentiation, effectively weakening the pro‐inflammatory response in both the PNS and CNS. The results support the efficaciousness of SVF therapy immunomodulation in EAE autoimmune and chronic inflammatory disease.

Though further validation is necessary, these studies offer compelling early evidence for SVF as a treatment modality for MS. The extent to which SVF induces immunomodulation is mediated by the favorable changes in the periphery, leading to the amelioration of pathology. In the course of the disease, Th1 and Th17 cells produce inflammatory cytokines, including IFN‐γ and IL‐12, that enable the differentiation of macrophages into the classical pro‐inflammatory phenotype.Pre‐clinical studies suggest that the SVF achieves its therapeutic impact by abrogating the inflammatory condition by reducing Th1 and Th17 frequency, which is associated with elevated alternative macrophage levels.^80^

To further unravel the impact of SVF‐mediated anti‐inflammatory induction, Th1 and T‐reg transcription factors were measured in splenocytes. SVF greatly reduced STAT1 while ASC treatment resulted in a modest decrease. STAT1 is an upstream regulator of Th1 differentiation and the decrease of STAT1 is associated with decreased IFNγ.^81^, ^82^, ^83^ SVF slightly reduced T‐bet transcription factor (implicated in Th1 differentiation) further supporting STAT1 reduction imparted by the SVF.

## 8 SVF‐MEDIATED INDUCTION OF ANTI‐INFLAMMATORY CYTOKINES

Studies suggest that SVF acts distally and systemically. The study performed by Semon et al. resulted in SVF‐induced suppression of MS/EAE pathogenesis‐related cytokines IL‐12 and IFN‐γ in the sera, indicative of a pathology‐attenuated response and reduced Th‐1 stimulation.^76^ Vehicle‐treated groups displayed significantly higher IL‐2 and IL‐12 levels in the sera compared to SVF‐ and ASC‐treated groups; IL‐2 stimulates T‐cell proliferation and this reduction may be responsible for thereduced Th1 T cellnumbers and immune reactivity in the spleen and lymph. Similarly, IFN‐γ levels were found undetectable in the SVF group while ASC treatment exhibited elevated IL‐6 and TNF levels. Unlike the spleen, TGFβ levels were detected at significantly lower amounts post‐SVF in the periphery.

## 9 IMPLICATION OF PRE‐CLINICAL FINDINGS FOR NEURODEGENERATIVE APPLICATIONS

Given the insidious progressive nature of neurodegenerative diseases, where continuous neurological deterioration occurs over decades, it is reasonable to assume that preventing early events of the immunological cascade would attenuate disease severity (Table 2). For MS, most immunotherapeutic drugs aim to resolve relapsing‐remitting MS, leaving progressive MS uncharted.^84^ Notably, immunotherapies are most effective when the clinical progression is predominantly immune‐mediated.^85^ Pre‐clinical studies of SVF administration resulted in the obstruction of chronic inflammation by hindering T‐reactive lymphocytes to stimulate immune tolerance, while repairing lesions and restoring motor and cognitive functions. We posit that the heterogeneous SVF composition synchronously communicates to resolve systemic and local aberrations by abrogating local and distal immune hyperactivity compared to the more conventional ASC or MSC purified counterparts.

**TABLE 2:** Review of available pre‐clinical studies using the stromal vascular fraction (SVF) for neurodegenerative diseases

PMID	SVF source	Disease	Administration route	Significant outcomes	Proposed mechanisms
29534751	Female GFP transgenic mice (C57Bl/6‐Tg(UBC‐GFP)30Scha/J strain, Jackson lab)	MS (modeled with early‐stage EAE)	IP injection of 10^6^ SVF cells/100 μl	Significant difference in disease severity by 30 DPI	‐ Concurrent reduction of IL‐2 compounds the effects of IL‐6 increase. ‐ Reduction in IFN, TNFα, IL‐4, IL‐12, and IL‐23 diminished number of encephalitogenic T cells. ‐ IL10 and TGF‐b increase induced differentiation of T cells to T regs
23981726	Male eGFP transgenic mice (C57Bl/6‐Tg(UBC‐GFP)30Scha/J strain; Jackson lab)	MS (modeled with EAE)	IP injection of 10^6^ SVF cells/100 μL	‐ SVF delayed disease onset (14 days) vs. control (9 days).	‐ Delayed disease onset due to reduction of IL‐12 and INF‐y which stifled the differentiation and expansion of Th1 subsets
27733015	Female eGFP transgenic mice (C57Bl/ 6‐Tg(UBC‐GFP)30Scha/J strain; Jackson Laboratory), ages 6–12 weeks	MS (modeled with late‐stage EAE)	IP injection of 10^6^ SVF cells/100 μL	‐ Average and cumulative disease severity scores significantly lower than controls at 10 DPI (30 DPI‐ EAE) ‐ Significant improvements in behaviour (wall leans, distance travelled in 5 min, average moving durations, average velocity of movement) ‐ Reduction of infiltrating immune cells and lesion frequency in spinal cord	‐ SVF enhanced IL‐10 secretion in the blood, brain and spinal cord, regulating T‐cell polarization to T regs and decreasing levels of cytolytic T cells
28801931	Male eGFP transgenic mice (transgenic mice (C57Bl/6‐Tg(UBC‐GFP)30Scha/J strain; Jackson Laboratory), age 2–6 months	MS (modeled with EAE)	IP injection of 10^6^ SVF cells/100 μL	‐ Decreased levels of immune infiltration in the spinal cord of EAE demonstrating an attenuation of neuroinflammation	‐ SVF alleviates disease leading to functional, immunological, and pathological improvements through the induction of T regs and alternative macrophages induction. ‐ Reduced Th1 and Th17 infiltration in the CNS resulted in correlative increases in IL‐10 levels in the spleen, suggesting that SVF impacts c.
24405805	Subcutaneous white adipose tissue of 3 human donors	MS (modelled with EAE)	IP injection of 10^6^ SVF cells/100 μl	‐ Mean maximum disease, cumulative disease and average disease severity scores were significantly reduced. ‐ Demyelination and myelin breakdown/debris was reduced in spinal cord.	‐ SVF provides therapeutic relief and ameliorates CNS damage in the early inflammatory phase.

*Note*: Review methods: PubMed – input words: SVF, stromal vascular fraction, neurodegeneration.

## 10 CLINICAL TRIALS OF SVF

### 10.1 SVF in MS

While SVF pre‐clinical data demonstrated potent therapeutic potential in chronic MS, understanding the mechanism by which the SVF influences the inflammatory milieu locally or peripherally is crucial to designing future clinical studies. The pending clinical trial of SVF for MS (GARM‐MS; NCT03461419; 100 study participants) employs a microcannula harvesting approach of autologous subdermal fat using an enzymatic digestion device (Centricyte 1000) for SVF isolation. Cells are deployed via intravascular routes in a sterile saline suspension (500 cc) as cells are small enough to migrate via the cerebral fluid and enter through defects of the blood‐brain barrier. This initial open‐label study aims to evaluate the safety of autologous SVF application and adverse reaction of systemically transplanted SVF cells in patients with progressive MS. A similar clinical intervention using autologous SVF applications aims to recruit patients for MS, autoimmune, inflammatory, and neurological conditions (NCT02939859; 100 study participants) with secondary outcomes evaluating fatigue and cognitive decline.

Similarly, a physician‐initiated case study for compassionate use explored adipose SVF in *n* = 3 MS patients in response to IV infusions of freshly extracted adipose‐derived SVF cells plus intrathecal and intravenous infusions of allogeneic culturally expanded CD34+ and MSCs. Treated patients experienced significant improvements in cognitive and motor function while two had no change in the size of the lesions as evidenced by MRI.^40^ The authors noted these improvements were not commonplace in past cases when only allogeneic CD34+ and MSC cells were used, implying that the nature of the recoveries observed resulted from the concomitant SVF administration. Further studies should explore the use of autologous SVF‐based therapies with varying constituents to understand how the composition of the applied cellular product impacts the extent of recovery.

### 10.2 Clinical trials for Parkinson's, Alzheimer's and ALS

A recent case study of two PD patients demonstrated sustained clinical improvement post‐treatment with facially transplanted adipose SVF. SVF utilized in the study was harvested from the patients’ subcutaneous adipose tissue and processed with either a GID‐SVF 1 or 2 devices.^86^ Patient 1 had a total yield of 300 cc of fat, containing 73.8 × 10^6^ TNC (total nucleated cell count) with 85% viability, while Patient 2 had a total output of 110 g of dry fat containing 68 × 10^6^ TNC with 90% viability. The cellular fraction was administered (100 cc Hartmann solution) in 0.5 cc aliquots to the superficial investing fascia submusculoaponeurotic fascia (Patient 1) and equally distributed between the superficial investing fascia (SMAS). Patient 2 received SVF treatment in the subperichondrial and subperiosteal planes of the nasal cartilage. This represents a novel delivery mechanism to the fascial tissue's blood‐rich regions, allowing cells to access neural circulation through venous drainage into ophthalmic and periorbital circulation.

Both patients under this trial saw marked improvements in clinical evaluations over 5 years (Patient 1) and 12 months (Patient 2). Similarly, the two patients saw clinically beneficial PDQ‐39 numeric scores decreased across all categories, especially in ‘mobility and activity of daily living’ compared to the expected score increase and symptoms worsening as further neurologic damage is incurred. Both patients also experienced decreasing severity scores (UPDRS) and motor scores while medication doses were maintained or reduced, in stark comparison to the expected progression of increasing motor scores redressed through increases in medication dosage. Interestingly, in both cases, the final ‘off’ medication (no dopaminergic or anticholinergics for >12 h) score was lower than their initial scores while on medication, demonstrating notable improvements in traditional evaluation metrics. The authors discuss the possibility of a potential long‐term activation mechanism acting through the presence of signaling cascades and cellular factors provoking trophic stimulation at the degree of the nigrostriatal pathway. This was supported by the earliest visibility of benefits at 2 weeks which then reached a maximum subjective effect at approximately 4–6 months and sustained for 5 years (Patient 1) and 12 months (Patient 2). The authors acknowledged the need for further investigation to delineate a mechanism of action. The significance of the recovery for these two patients warrants deeper exploration.

A 3‐year Phase I clinical trial recently explored the treatment potential and safety of human intracerebroventricular (ICV) brain injections of SVF in patients suffering from a neurodegenerative disease wherein no other treatment option is available.^87^ The study enrolled *n* = 24 patients who received SVF into the frontal horn of the lateral ventricle using the Ommaya reservoir, and *n* = 6 received injections through the ventriculo‐peritoneal shafts; the median patient age was 74 years old, ranging from 41 to 83 years of age. Among the patients tested, *n* = 10 had Alzheimer's, *n* = 6 had ALS, *n* = 6 had multiple sclerosis (MS‐P) and *n* = 6 had Parkinson's ‘Plus’ (PD+). The composition of the 3.5–20 cc SVF injections were, on average, 8% haemopoietic stem cells and 7.5 % adipose‐derived stem cells. Among the patients with Alzheimer's, 80% remained stable or demonstrated improvements in cognitive tests, while 20% displayed improvements in p‐tau and ß‐amyloid levels. Secondary endpoints showed stability with limited progress in the MS and AD patients, suggesting that the SVF reduces disease propagation across neurodegenerative diseases.

To further this study, authors conducted pre‐SVF and inter‐SVF NeuroQuant volumetric analyses on 40% of AD patients and concluded that one patient displayed an increase in hippocampal volume. However, a closer observation of the MRI images exhibited ambiguity to conclusively claim hippocampal volume increase. For AD, the authors discuss a general stabilization or increase in MPI (Memory Performance Index) for *n* = 4 patients compared to a ‘typical’ AD patient (study does not define ‘typical’ parameters). Therefore, caution must be taken when comparing the MPI performance of patients at various stages in their disease to a ‘typical’ Alzheimer's patient, which does not exist at the level of definition insinuated; a smooth curve decline as ‘typical’ decline is most probably not an accurate descriptor for these measurements taken at discrete time‐points and therefore the comparisons lack substantiated evidence. Further studies must ensure adequate standardized methodology to measure therapeutic outcomes across patients.

For ALS, 33% of patients remained stable, and 66% passed due to disease progression. Of patients with Parkinson's, three died of natural disease progression, one withdrew after symptoms worsened following a singular dose, and two remained stable. Of the six MS‐P patients, all remained stable or demonstrated improvements.

In the mentioned clinical trial, MS‐P and AD patients experienced a ‘wearing down’ of effects around 6–8 weeks post‐treatment; however, as the number of injections increased, this effect was anecdotally decreased. The study therefore concluded l that repeated treatments may illicit recovery of neurons and prolonged initial anti‐inflammatory response. There were 113 injections administered; 11% led to 1–4 days of transient meningismus and moderate temperature increase alleviated by acetaminophen and dexamethasone. Only two injections required hospitalization for these symptoms. The injections were considered a safe treatment option for patients with no alternative recourse. Given such promise, the results from this study are now pending submission for a Phase II clinical trial.

Finally, a clinical trial evaluating neurological and non‐neoplastic disorders and diseases using autologous subdermal SVF isolation is underway. An open‐label, single‐group, interventional study, set to recruit 300 participants (NCT03297177), evaluates the safety and efficacy of adipose‐derived SVF. Subdermal fat is isolated through a digestive process and pelleted through standard centrifugation, wherein SVF cells will be suspended with saline solution (500 ccs) and re‐administered via intravascular injection. Patients receiving this intervention must present neurological damage to the central or peripheral nervous system and be non‐responsive to current standard of care approaches.

Overall, it is unclear how or why some patients respond better to SVF while others were unaffected; possible reasons could be due to differences in SVF composition across patients. Standardized clinical trial outcomes using SVF may emerge from identifying therapeutically responsible constituents in SVF across patient composition; nonetheless, these clinical trials suggest that SVF is a promising autologous modality to mitigate inflammation in patients for which there is no other recourse (Table 3).

**TABLE 3:** Human clinical application using the stromal vascular fraction (SVF) with outcomes

Trial	Title	Diseases	SVF concentration and administration route
31327120	Human intracerebroventricular (ICV) injection of autologous, non‐engineered, adipose‐derived stromal vascular fraction (ADSVF) for neurodegenerative disorders: results of a 3‐year Phase I study of 113 injections in 31 patients	‐ Amyotrophic lateral sclerosis (ALS), *n* = 6 ‐ Dementias (AD), *n* = 10 ‐ Parkinson's‐like syndromes (PD+) (including multiple system atrophy and progressive supranuclear palsy, *n* = 6 ‐ Traumatic brain injury (TBI), *n* = 1 ‐ Multiple sclerosis—progressive form (MS‐P), *n* = 6 ‐ Stroke, *n* = 1 ‐ Spinal cord injury (SCI), *n* = 1	‐ Frontal horn of lateral ventricle via Ommaya reservoir (*n* = 24) ‐ shunt (*n* = 7) ‐ 4.05 × 10^5^–6.2 × 10^7^ cells/cc ‐ 3.5–20 cc injected (median 4 cc)
33222965	Sustained clinical improvement of Parkinson's disease in two patients with facially transplanted adipose‐derived stromal vascular fraction cells	‐ Parkinson's	Facial injections using 21 g needle ‐ Patient 1: 73.8 × 10^6^ cells suspended in 100 cc of Hartmann solution, distributed equally in 3 different locations ‐ Patient 2: 68 × 10^6^ cells suspended in 100 cc of Hartmann solution, distributed equally between 2 sites

*Note*: Review methods: PubMed and clinicaltrial.gov search – input words: SVF, stromal vascular fraction, neurodegeneration.

## 11 FUTURE DIRECTION

Due to SVF's heterogeneous nature, identifying biological factors influencing therapeutic outcomes can inform patient selection and dosing. While SVF maintains a relatively high safety index across all pre‐clinical and clinical studies, administration at different time‐points during disease progression or evaluation of engraftment and retention in situ can augment understanding of local and systemic impacts. Though intraperitoneal injections resulted in systemic alterations in the spleen, lymphatic organs and blood, local applications to the CNS through reservoirs or catheters in pre‐clinical models are necessary to fully comprehend its therapeutic potency. Furthermore, point‐of‐care isolation methods are expected to produce different outcomes, though the extent is unknown. Hence, variability of patient composition may reflect, in part, the different mechanisms by which SVF imparts its benefits. To improve understanding, analysis of cellular populations across SVF profiles will help predict the influence of certain constituents in SVF and across studies. To enhance the identification of therapeutically essential components in SVF, composition based on surface markers (through flow cytometry, or cytometry time of flight) should be standardized and must precede administration. This will allow a more divisive approach to patient selection as we attempt to comprehend the full spectrum of SVF therapeutic potency across different diseases. Additionally, collection of biological metrics (weight, age, sex, and co‐morbidities) will aid in identifying risk factors that may enhance or diminish SVF treatment outcomes and potency.

**FIGURE 3:**
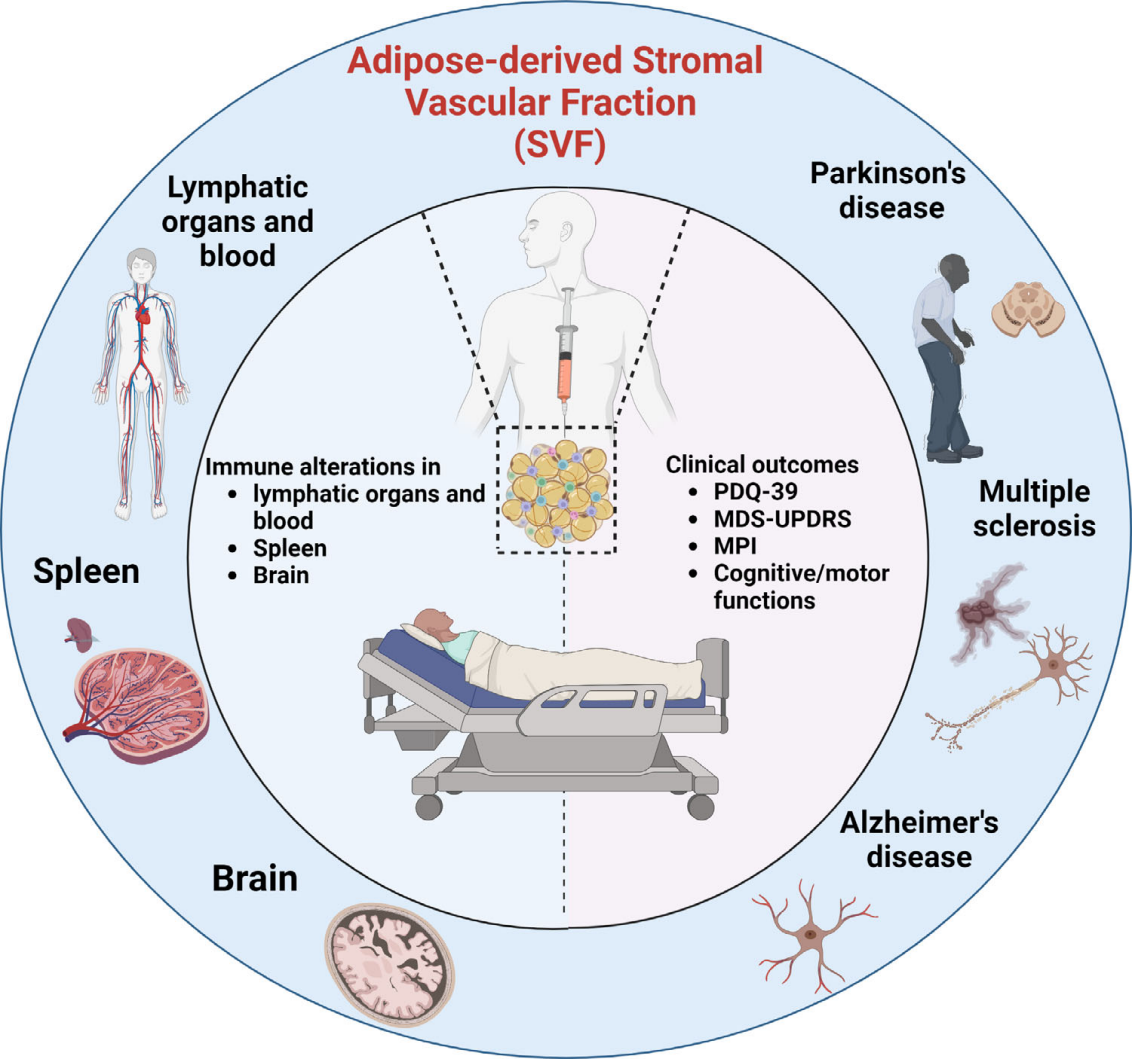
SVF clinical application for neurodegenerative diseases. PDQ‐39: Parkinson's Disease Questionnaire; MDS‐UPDRS: Movement Disorder Society Unified Parkinson's Disease Rating Scale; MPI: Memory Performance Index

Clinically, neurodegenerative diseases are diagnosed and monitored using several criteria. The impact of SVF therapy can be measured using disease‐specific clinical metrics such as the expanded disability status scale (EDSS) for MS or the Movement Disorder Society Unified Disease Rating Scale (MDS‐UPDRS) for PD, or tracking of tau or amyloid through positron emission tomography (PET) for AD.^88^ To supplement clinical monitoring, identifying a therapeutic axis or mechanisms using molecular surrogates beyond functional and clinical recovery endpoints (cytokines, alterations in immune infiltrates, biomarkers in cerebrospinal fluid, changes to PBMC) would support the mounds of clinical data associated with disease response (Figure 3).

## 12 CONCLUSIONS

Cell‐based therapies for all conditions (except for hematopoietic transplants) are still experimental. The novel application of ‘at point of care’ cell therapy (wherein the cellular product is isolated, modified and applied same day) may be hindered by traditional regulatory guidelines designed for stem cells requiring long‐term manufacturing, trained personnel, specialized equipment for expansion, sterility, long‐term monitoring and concerns of product identity and purity as a consequence of manufacturing. While the regulatory hurdles on traditional cell therapy applications requiring long‐term assessments protect ethical, moral and safety guidelines, alternative applications (such as the autologous and same‐day use of SVF) may be overlooked due to historical guidelines originally being designed with long‐term manufacturing of purified cellular products in mind.

Patients often present with diverse manifestations of the same disease. As we advance clinical care and treatment paradigms, taking advantage of the natural repair mechanisms cellular therapy inherently provides can be beneficial. This is especially relevant for an aging population where repair mechanisms remain dormant or the healing process requires multiple paradigms for correction. Importantly, because the SVF possesses all the relevant components (immune, stem and progenitor cells) in one fraction, it can actively intake information from the environment (site of implantation) and engage in bi‐directional communication to mediate homeostasis according to local tissue needs with early clinical evidence pointing to applications in other pathological contexts, such as cancer.^89^

SVF stands as an exciting and emerging cellular therapeutic, warranting further investigation. Evidence suggests that SVF owes its therapeutic benefits to its immunomodulatory mechanisms rather than the propensity of its constituents to differentiate and repair aberrant cells. It is conceivable to attribute paracrine signaling as the mode of action rather than cell retention and engraftment in the tissue, a presumption and rationale associated with other cell therapies. Benefits may be mediated through soluble factors that facilitate site‐specific tissue remodeling in damaged lesions through T‐cell differentiation, macrophage polarization, and cytokine secretion locally and systemically. Hence, a key component in advancing regenerative medicine may be the administration of a physiologically relevant cellular milieu, as seen in SVF, able to withstand a metabolically taxing process such as ‘cell engraftment’. The synchronous application of immune, stem and stromal cells offers an alternative therapy by enhancing reparative mechanisms in situ.

## FUNDING

Center for Regenerative Medicine Research Grant, Mayo Clinic.

## CONFLICT OF INTEREST

No conflict of interests or financial affiliations to disclose.

## References

[1] Yang W , Hamilton JL , Kopil C , et al. Current and projected future economic burden of Parkinson's disease in the U.S. NPJ Parkinsons Dis. 2020;6:15. doi: 10.1038/s41531-020-0117-1 32665974 PMC7347582

[2] Gooch CL , Pracht E , Borenstein AR . The burden of neurological disease in the United States: a summary report and call to action. Ann Neurol. 2017;81:479‐484. doi: 10.1002/ana.24897 28198092

[3] Wong W . Economic burden of Alzheimer disease and managed care considerations. Am J Manag Care. 2020;26:S177‐s183. doi:10.37765/ajmc.2020.88482 32840331

[4] Bejot Y , Yaffe K . Ageing population: a neurological challenge. Neuroepidemiology. 2019;52:76‐77. doi:10.1159/000495813 30602150

[5] Amor S , Puentes F , Baker D , van der Valk P . Inflammation in neurodegenerative diseases. Immunology. 2010;129:154‐169. doi:10.1111/j.1365-2567.2009.03225.x 20561356 PMC2814458

[6] Chen GY , Nunez G . Sterile inflammation: sensing and reacting to damage. Nat Rev Immunol. 2010;10:826‐837. doi:10.1038/nri2873 21088683 PMC3114424

[7] Lunn JS , Sakowski SA , Hur J , Feldman EL . Stem cell technology for neurodegenerative diseases. Ann Neurol. 2011;70:353‐361. doi:10.1002/ana.22487 21905078 PMC3177143

[8] Lee IS , Jung K , Kim IS , et al. Human neural stem cells alleviate Alzheimer‐like pathology in a mouse model. Mol Neurodegener. 2015;10:38. doi:10.1186/s13024-015-0035-6 26293123 PMC4546205

[9] Mendes‐Pinheiro B , Teixeira FG , Anjo SI , Manadas B , Behie LA , Salgado AJ . Secretome of undifferentiated neural progenitor cells induces histological and motor improvements in a rat model of Parkinson's disease. Stem Cells Transl Med. 2018;7:829‐838. doi:10.1002/sctm.18-0009 30238668 PMC6216452

[10] Redmond DE Jr , Bjugstad KB , Teng YD , et al. Behavioral improvement in a primate Parkinson's model is associated with multiple homeostatic effects of human neural stem cells. Proc Natl Acad Sci U S A. 2007;104:12175‐12180. doi:10.1073/pnas.0704091104 17586681 PMC1896134

[11] McGinley LM , Sims E , Lunn JS , et al. Human cortical neural stem cells expressing insulin‐like growth factor‐I: a novel cellular therapy for Alzheimer's disease. Stem Cells Transl Med. 2016;5:379‐391. doi:10.5966/sctm.2015-0103 26744412 PMC4807660

[12] Blurton‐Jones M , Kitazawa M , Martinez‐Coria H , et al. Neural stem cells improve cognition via BDNF in a transgenic model of Alzheimer disease. Proc Natl Acad Sci U S A. 2009;106:13594‐13599. doi:10.1073/pnas.0901402106 19633196 PMC2715325

[13] Ager RR , Davis JL , Agazaryan A , et al. Human neural stem cells improve cognition and promote synaptic growth in two complementary transgenic models of Alzheimer's disease and neuronal loss. Hippocampus. 2015;25:813‐826. doi:10.1002/hipo.22405 25530343 PMC4722865

[14] Hu BY , Weick JP , Yu J , et al. Neural differentiation of human induced pluripotent stem cells follows developmental principles but with variable potency. Proc Natl Acad Sci U S A. 2010;107:4335‐4340. doi:10.1073/pnas.0910012107 20160098 PMC2840097

[15] Dimos JT , Rodolfa KT , Niakan KK , et al. Induced pluripotent stem cells generated from patients with ALS can be differentiated into motor neurons. Science. 2008;321:1218‐1221. doi:10.1126/science.1158799 18669821

[16] Wernig M , Zhao JP , Pruszak J , et al. Neurons derived from reprogrammed fibroblasts functionally integrate into the fetal brain and improve symptoms of rats with Parkinson's disease. Proc Natl Acad Sci U S A. 2008;105:5856‐5861. doi:10.1073/pnas.0801677105 18391196 PMC2311361

[17] Xiong N , Zhang Z , Huang J , et al. VEGF‐expressing human umbilical cord mesenchymal stem cells, an improved therapy strategy for Parkinson's disease. Gene Ther. 2011;18:394‐402. doi:10.1038/gt.2010.152 21107440

[18] Bae JS , Jin HK , Lee JK , Richardson JC , Carter JE . Bone marrow‐derived mesenchymal stem cells contribute to the reduction of amyloid‐beta deposits and the improvement of synaptic transmission in a mouse model of pre‐dementia Alzheimer's disease. Curr Alzheimer Res. 2013;10:524‐531. 23036020

[19] Ma T , Gong K , Ao Q , et al. Intracerebral transplantation of adipose‐derived mesenchymal stem cells alternatively activates microglia and ameliorates neuropathological deficits in Alzheimer's disease mice. Cell Transplant. 2013;22(Suppl 1):S113‐26. doi:10.3727/096368913X672181 24070198

[20] Mostafavi H , Ghassemifard L , Rostami A , Alipour M , Nadri S . Trabecular meshwork mesenchymal stem cell transplantation improve motor symptoms of parkinsonian rat model. Biologicals. 2019;61:61‐67. doi:10.1016/j.biologicals.2019.06.006 31262640

[21] Dykstra JA , Facile T , Patrick RJ , et al. Concise review: fat and furious: harnessing the full potential of adipose‐derived stromal vascular fraction. Stem Cells Transl Med. 2017;6:1096‐1108. doi:10.1002/sctm.16-0337 28186685 PMC5388064

[22] Allgaier M , Allgaier C . An update on drug treatment options of Alzheimer's disease. Front Biosci (Landmark Ed). 2014;19:1345‐1354. doi:10.2741/4285 24896354

[23] Mehta D , Jackson R , Paul G , Shi J , Sabbagh M . Why do trials for Alzheimer's disease drugs keep failing? A discontinued drug perspective for 2010–2015. Expert Opin Investig Drugs. 2017;26:735‐739. doi:10.1080/13543784.2017.1323868 PMC5576861 28460541

[24] Petrov D , Mansfield C , Moussy A , Hermine O . ALS clinical trials review: 20 years of failure. Are we any closer to registering a new treatment? Front Aging Neurosci. 2017;9:68. doi:10.3389/fnagi.2017.00068 28382000 PMC5360725

[25] De Gioia R , Biella F , Citterio G , et al. Neural stem cell transplantation for neurodegenerative diseases. Int J Mol Sci. 2020;21. doi:10.3390/ijms21093103 PMC7247151 32354178

[26] Ramakrishnan VM , Boyd NL . The adipose stromal vascular fraction as a complex cellular source for tissue engineering applications. Tissue Eng Part B Rev. 2018;24:289‐299. doi:10.1089/ten.TEB.2017.0061 28316259 PMC6080106

[27] Karantalis V , Schulman IH , Balkan W , Hare JM . Allogeneic cell therapy: a new paradigm in therapeutics. Circ Res. 2015;116:12‐15. doi:10.1161/CIRCRESAHA.114.305495 25552688 PMC4411634

[28] Duinsbergen D , Salvatori D , Eriksson M , Mikkers H . Tumors originating from induced pluripotent stem cells and methods for their prevention. Ann N Y Acad Sci. 2009;1176:197‐204. doi:10.1111/j.1749-6632.2009.04563.x 19796248

[29] Li JY , Englund E , Holton JL , et al. Lewy bodies in grafted neurons in subjects with Parkinson's disease suggest host‐to‐graft disease propagation. Nat Med. 2008;14:501‐503. doi:10.1038/nm1746 18391963

[30] Seminatore C , Polentes J , Ellman D , et al. The postischemic environment differentially impacts teratoma or tumor formation after transplantation of human embryonic stem cell‐derived neural progenitors. Stroke. 2010;41:153‐159. doi:10.1161/STROKEAHA.109.563015 19940279

[31] Lo B , Parham L . Resolving ethical issues in stem cell clinical trials: the example of Parkinson disease. J Law Med Ethics. 2010;38:257‐266. doi:10.1111/j.1748-720X.2010.00486.x 20579249

[32] Marconi S , Bonaconsa M , Scambi I , et al. Systemic treatment with adipose‐derived mesenchymal stem cells ameliorates clinical and pathological features in the amyotrophic lateral sclerosis murine model. Neuroscience. 2013;248:333‐343. doi:10.1016/j.neuroscience.2013.05.034 23727509

[33] Zangi L , Margalit R , Reich‐Zeliger S , et al. Direct imaging of immune rejection and memory induction by allogeneic mesenchymal stromal cells. Stem Cells. 2009;27:2865‐2874. doi:10.1002/stem.217 19750539

[34] Guo Y , Su L , Wu J , et al. Assessment of the green florescence protein labeling method for tracking implanted mesenchymal stem cells. Cytotechnology. 2012;64:391‐401. doi:10.1007/s10616-011-9417-y 22373822 PMC3397108

[35] Takahashi K , Yamanaka S . Induction of pluripotent stem cells from mouse embryonic and adult fibroblast cultures by defined factors. Cell. 2006;126:663‐676. doi:10.1016/j.cell.2006.07.024 16904174

[36] Bora P , Majumdar AS . Adipose tissue‐derived stromal vascular fraction in regenerative medicine: a brief review on biology and translation. Stem Cell Res Ther. 2017;8:145. doi:10.1186/s13287-017-0598-y 28619097 PMC5472998

[37] Bourin P , Bunnell BA , Casteilla L , et al. Stromal cells from the adipose tissue‐derived stromal vascular fraction and culture expanded adipose tissue‐derived stromal/stem cells: a joint statement of the International Federation for Adipose Therapeutics and Science (IFATS) and the International Society for Cellular Therapy (ISCT). Cytotherapy. 2013;15:641‐648. doi:10.1016/j.jcyt.2013.02.006 23570660 PMC3979435

[38] Koh YJ , Koh BI , Kim H , et al. Stromal vascular fraction from adipose tissue forms profound vascular network through the dynamic reassembly of blood endothelial cells. Arterioscler Thromb Vasc Biol. 2011;31:1141‐1150. doi:10.1161/ATVBAHA.110.218206 21393582

[39] Morris ME , Beare JE , Reed RM , et al. Systemically delivered adipose stromal vascular fraction cells disseminate to peripheral artery walls and reduce vasomotor tone through a CD11b+ cell‐dependent mechanism. Stem Cells Transl Med. 2015;4:369‐380. doi:10.5966/sctm.2014-0252 25722428 PMC4367510

[40] Riordan NH , Ichim TE , Min WP , et al. Non‐expanded adipose stromal vascular fraction cell therapy for multiple sclerosis. J Transl Med. 2009;7:29. doi:10.1186/1479-5876-7-29 19393041 PMC2679713

[41] Dong Z , Fu R , Liu L , Lu F . Stromal vascular fraction (SVF) cells enhance long‐term survival of autologous fat grafting through the facilitation of M2 macrophages. Cell Biol Int. 2013;37:855‐859. doi:10.1002/cbin.10099 23526646

[42] Sahin U , Tureci O . Personalized vaccines for cancer immunotherapy. Science. 2018;359:1355‐1360. doi:10.1126/science.aar7112 29567706

[43] Park IH , Arora N , Huo H , et al. Disease‐specific induced pluripotent stem cells. Cell. 2008;134:877‐886. doi:10.1016/j.cell.2008.07.041 18691744 PMC2633781

[44] Hanna JH , Saha K , Jaenisch R . Pluripotency and cellular reprogramming: facts, hypotheses, unresolved issues. Cell. 2010;143:508‐525. doi:10.1016/j.cell.2010.10.008 21074044 PMC3032267

[45] Sulzer D , Alcalay RN , Garretti F , et al. Erratum: t cells from patients with Parkinson's disease recognize alpha‐synuclein peptides. Nature. 2017;549:292. doi:10.1038/nature23896 PMC10204083 28905919

[46] Franceschi C , Bonafè M , Valensin S , et al. Inflamm‐aging. An evolutionary perspective on immunosenescence. Ann N Y Acad Sci. 2000;908:244‐254. doi:10.1111/j.1749-6632.2000.tb06651.x 10911963

[47] Rohn TT . Cytoplasmic inclusions of TDP‐43 in neurodegenerative diseases: a potential role for caspases. Histol Histopathol. 2009;24:1081‐1086. doi: 10.14670/HH-24.1081 19554515 PMC2791961

[48] Stone S , Lin W . The unfolded protein response in multiple sclerosis. Front Neurosci. 2015;9:264. doi:10.3389/fnins.2015.00264 26283904 PMC4518158

[49] Xu L , Pu J . Alpha‐synuclein in Parkinson's disease: from pathogenetic dysfunction to potential clinical application. Parkinsons Dis. 2016;2016:1720621. doi:10.1155/2016/1720621 27610264 PMC5005546

[50] Ishizawa T , Mattila P , Davies P , Wang D , Dickson DW . Colocalization of tau and alpha‐synuclein epitopes in Lewy bodies. J Neuropathol Exp Neurol. 2003;62:389‐397. doi:10.1093/jnen/62.4.389 12722831

[51] Hardy J , Selkoe DJ . The amyloid hypothesis of Alzheimer's disease: progress and problems on the road to therapeutics. Science. 2002;297:353‐356. doi:10.1126/science.1072994 12130773

[52] Selkoe DJ . Cell biology of protein misfolding: the examples of Alzheimer's and Parkinson's diseases. Nat Cell Biol. 2004;6:1054‐1061. doi:10.1038/ncb1104-1054 15516999

[53] Lucchinetti C , Bruck W , Parisi J , Scheithauer B , Rodriguez M , Lassmann H . Heterogeneity of multiple sclerosis lesions: implications for the pathogenesis of demyelination. Ann Neurol. 2000;47:707‐717. doi:10.1002/1531-8249(200006)47:6<707::aid-ana3>3.0.co;2-q 10852536

[54] Frohman EM , Racke MK , Raine CS . Multiple sclerosis–the plaque and its pathogenesis. N Engl J Med. 2006;354:942‐955. doi:10.1056/NEJMra052130 16510748

[55] Machado‐Santos J , Saji E , Troscher AR , et al. The compartmentalized inflammatory response in the multiple sclerosis brain is composed of tissue‐resident CD8+ T lymphocytes and B cells. Brain. 2018;141:2066‐2082. doi:10.1093/brain/awy151 29873694 PMC6022681

[56] Ortiz GG , Pacheco‐Moises FP , Macias‐Islas MA , et al. Role of the blood‐brain barrier in multiple sclerosis. Arch Med Res. 2014;45:687‐697. doi:10.1016/j.arcmed.2014.11.013 25431839

[57] Jafarzadeh Bejargafshe M, Hedayati M , Zahabiasli S , Tahmasbpour E , Rahmanzadeh S , Nejad‐Moghaddam A . Safety and efficacy of stem cell therapy for treatment of neural damage in patients with multiple sclerosis. Stem Cell Investig. 2019;6:44. doi: 10.21037/sci.2019.10.06 PMC6987330 32039266

[58] Zufiría M , Gil‐Bea FJ , Fernández‐Torrón R , et al. ALS: a bucket of genes, environment, metabolism and unknown ingredients. Prog Neurobiol. 2016;142:104‐129. doi:10.1016/j.pneurobio.2016.05.004 27236050

[59] Mitsumoto H , Brooks BR , Silani V . Clinical trials in amyotrophic lateral sclerosis: why so many negative trials and how can trials be improved? Lancet Neurol. 2014;13:1127‐1138. doi:10.1016/s1474-4422(14)70129-2 25316019

[60] DeLoach A , Cozart M , Kiaei A , Kiaei M . A retrospective review of the progress in amyotrophic lateral sclerosis drug discovery over the last decade and a look at the latest strategies. Expert Opin Drug Discov. 2015;10:1099‐1118. doi:10.1517/17460441.2015.1067197 26307158

[61] Tortelli R , Zecca C , Piccininni M , et al. Plasma inflammatory cytokines are elevated in ALS. Front Neurol. 2020;11:552295. doi:10.3389/fneur.2020.552295 33281700 PMC7691268

[62] Smith JA , Das A , Ray SK , Banik NL . Role of pro‐inflammatory cytokines released from microglia in neurodegenerative diseases. Brain Res Bull. 2012;87:10‐20. doi:10.1016/j.brainresbull.2011.10.004 22024597 PMC9827422

[63] Barger SW , Harmon AD . Microglial activation by Alzheimer amyloid precursor protein and modulation by apolipoprotein E. Nature. 1997;388:878‐881. doi:10.1038/42257 9278049

[64] Dugan LL , Ali SS , Shekhtman G , et al. IL‐6 mediated degeneration of forebrain GABAergic interneurons and cognitive impairment in aged mice through activation of neuronal NADPH oxidase. PLoS One. 2009;4:e5518. doi:10.1371/journal.pone.0005518 19436757 PMC2678193

[65] Wang DB , Dayton RD , Zweig RM , Klein RL . Transcriptome analysis of a tau overexpression model in rats implicates an early pro‐inflammatory response. Exp Neurol. 2010;224:197‐206. doi:10.1016/j.expneurol.2010.03.011 20346943 PMC2906769

[66] Ghosh A , Roy A , Liu X , et al. Selective inhibition of NF‐kappaB activation prevents dopaminergic neuronal loss in a mouse model of Parkinson's disease. Proc Natl Acad Sci U S A. 2007;104:18754‐18759. doi:10.1073/pnas.0704908104 18000063 PMC2141849

[67] Wilms H , Rosenstiel P , Sievers J , Deuschl G , Zecca L , Lucius R . Activation of microglia by human neuromelanin is NF‐kappaB dependent and involves p38 mitogen‐activated protein kinase: implications for Parkinson's disease. FASEB J. 2003;17:500‐502. doi:10.1096/fj.02-0314fje 12631585

[68] Jaworski T , Lechat B , Demedts D , et al. Dendritic degeneration, neurovascular defects, and inflammation precede neuronal loss in a mouse model for tau‐mediated neurodegeneration. Am J Pathol. 2011;179:2001‐2015. doi:10.1016/j.ajpath.2011.06.025 21839061 PMC3181369

[69] Ding W , Ding LJ , Li FF , Han Y , Mu L . Neurodegeneration and cognition in Parkinson's disease: a review. Eur Rev Med Pharmacol Sci. 2015;19:2275‐2281. 26166654

[70] Brown RG , Marsden CD . Internal versus external cues and the control of attention in Parkinson's disease. Brain. 1988;111(Pt 2):323‐345. doi:10.1093/brain/111.2.323 3378139

[71] Bowen FP , Hoehn MM , Yahr MD . Parkinsonism: alterations in spatial orientation as determined by a route‐walking test. Neuropsychologia. 1972;10:355‐361. doi:10.1016/0028-3932(72)90027-9 5080496

[72] Oertel W , Schulz JB . Current and experimental treatments of Parkinson disease: a guide for neuroscientists. J Neurochem. 2016;139(Suppl 1):325‐337. doi:10.1111/jnc.13750 27577098

[73] Imamura K , Hishikawa N , Sawada M , Nagatsu T , Yoshida M , Hashizume Y . Distribution of major histocompatibility complex class II‐positive microglia and cytokine profile of Parkinson's disease brains. Acta Neuropathol. 2003;106:518‐526. doi:10.1007/s00401-003-0766-2 14513261

[74] Zappia E , Casazza S , Pedemonte E , et al. Mesenchymal stem cells ameliorate experimental autoimmune encephalomyelitis inducing T‐cell anergy. Blood. 2005;106:1755‐1761. doi:10.1182/blood-2005-04-1496 15905186

[75] Park JB , Lee JS , Cho BP , et al. Adipose tissue‐derived mesenchymal stem cells cultured at high cell density express brain‐derived neurotrophic factor and exert neuroprotective effects in a 6‐hydroxydopamine rat model of Parkinson's disease. Genes Genomics. 2015;37:213‐221. doi:10.1007/s13258-014-0239-0

[76] Semon JA , Zhang X , Pandey AC , et al. Administration of murine stromal vascular fraction ameliorates chronic experimental autoimmune encephalomyelitis. Stem Cells Transl Med. 2013;2:789‐796. doi:10.5966/sctm.2013-0032 23981726 PMC3785263

[77] Semon JA , Maness C , Zhang X , et al. Comparison of human adult stem cells from adipose tissue and bone marrow in the treatment of experimental autoimmune encephalomyelitis. Stem Cell Res Ther. 2014;5:2. doi:10.1186/scrt391 24405805 PMC4054950

[78] Bowles AC , Wise RM , Gerstein BY , et al. Adipose stromal vascular fraction attenuates T(H)1 cell‐mediated pathology in a model of multiple sclerosis. J Neuroinflammation. 2018;15:77. doi:10.1186/s12974-018-1099-3 29534751 PMC5850918

[79] Bowles AC , Strong AL , Wise RM , et al. Adipose stromal vascular fraction‐mediated improvements at late‐stage disease in a murine model of multiple sclerosis. Stem Cells. 2017;35:532‐544. doi:10.1002/stem.2516 27733015

[80] Bowles AC , Wise RM , Gerstein BY , et al. Immunomodulatory effects of adipose stromal vascular fraction cells promote alternative activation macrophages to repair tissue damage. Stem Cells. 2017;35:2198‐2207. doi:10.1002/stem.2689 28801931

[81] Ng TH , Britton GJ , Hill EV , Verhagen J , Burton BR , Wraith DC , (2013) Regulation of adaptive immunity; the role of interleukin‐10. Front Immunol. 4:129. doi:10.3389/fimmu.2013.00129 PMC3668291 23755052

[82] Lazarevic V , Glimcher LH , Lord GM . T‐bet: a bridge between innate and adaptive immunity. Nat Rev Immunol. 2013;13:777‐789. doi:10.1038/nri3536 24113868 PMC6290922

[83] Liao W , Lin JX , Leonard WJ . IL‐2 family cytokines: new insights into the complex roles of IL‐2 as a broad regulator of T helper cell differentiation. Curr Opin Immunol. 2011;23:598‐604. doi:10.1016/j.coi.2011.08.003 21889323 PMC3405730

[84] Lassmann H , van Horssen J , Mahad D . Progressive multiple sclerosis: pathology and pathogenesis. Nat Rev Neurol. 2012;8:647‐656. doi:10.1038/nrneurol.2012.168 23007702

[85] Coret F , Pérez‐Miralles FC , Gascón F , et al. Onset of secondary progressive multiple sclerosis is not influenced by current relapsing multiple sclerosis therapies. Mult Scler J Exp Transl Clin. 2018;4:2055217318783347. doi:10.1177/2055217318783347 30090637 PMC6077906

[86] Carstens M , Haq I , Martinez‐Cerrato J , Dos‐Anjos S , Bertram K , Correa D . Sustained clinical improvement of Parkinson's disease in two patients with facially‐transplanted adipose‐derived stromal vascular fraction cells. J Clin Neurosci. 2020;81:47‐51. doi:10.1016/j.jocn.2020.09.001 33222965

[87] Duma C , Kopyov O , Kopyov A , et al. Human intracerebroventricular (ICV) injection of autologous, non‐engineered, adipose‐derived stromal vascular fraction (ADSVF) for neurodegenerative disorders: results of a 3‐year phase 1 study of 113 injections in 31 patients. Mol Biol Rep. 2019;46:5257‐5272. doi:10.1007/s11033-019-04983-5 31327120

[88] Cotta Ramusino M , Perini G , Altomare D , et al. Outcomes of clinical utility in amyloid‐PET studies: state of art and future perspectives. Eur J Nucl Med Mol Imaging. 2021;48:2157‐2168. doi:10.1007/s00259-020-05187-x 33594474 PMC8175294

[89] Minev BR , Lander E , Feller JF , et al. First‐in‐human study of TK‐positive oncolytic vaccinia virus delivered by adipose stromal vascular fraction cells. J Transl Med. 2019;17:271. doi:10.1186/s12967-019-2011-3 31426803 PMC6699108

[90] Potential fabrication in research images threatens key theory of Alzheimer’s disease. 2022. AAAS Articles DO Group. doi:10.1126/science.ade0209

